# CT-Guided Transthoracic Core-Needle Biopsy of Pulmonary Nodules: Current Practices, Efficacy, and Safety Considerations

**DOI:** 10.3390/jcm13237330

**Published:** 2024-12-02

**Authors:** Amalia Constantinescu, Emil Robert Stoicescu, Roxana Iacob, Cosmin Alexandru Chira, Daiana Marina Cocolea, Alin Ciprian Nicola, Roxana Mladin, Cristian Oancea, Diana Manolescu

**Affiliations:** 1 Doctoral School, ‘Victor Babes’ University of Medicine and Pharmacy Timisoara, Eftimie Murgu Square 6 No. 2, 300041 Timisoara, Romania; amalia.constantinescu@umft.ro (A.C.); cosmin.chira@umft.ro (C.A.C.); daiana.cocolea@umft.ro (D.M.C.); alin.nicola@umft.ro (A.C.N.); roxana.mladin@umft.ro (R.M.); 2 Radiology and Medical Imaging University Clinic, Department XV, ‘Victor Babes’ University of Medicine and Pharmacy Timisoara, Eftimie Murgu Square No. 2, 300041 Timisoara, Romania; dmanolescu@umft.ro; 3 Research Center for Medical Communication, ‘Victor Babes’ University of Medicine and Pharmacy Timisoara, Eftimie Murgu Square No. 2, 300041 Timisoara, Romania; roxana.iacob@umft.ro; 4 Research Center for Pharmaco-Toxicological Evaluations, ‘Victor Babes’ University of Medicine and Pharmacy Timisoara, Eftimie Murgu Square No. 2, 300041 Timisoara, Romania; 5 Field of Applied Engineering Sciences, Specialization Statistical Methods and Techniques in Health and Clinical Research, Faculty of Mechanics, ‘Politehnica’ University Timisoara, Mihai Viteazul Boulevard No. 1, 300222 Timisoara, Romania; 6 Department of Anatomy and Embryology, ‘Victor Babes’ University of Medicine and Pharmacy Timisoara, Eftimie Murgu Square No. 2, 300041 Timisoara, Romania; 7 Center for Research and Innovation in Precision Medicine of Respiratory Diseases (CRIPMRD), ‘Victor Babes’ University of Medicine and Pharmacy Timisoara, 300041 Timisoara, Romania; oancea@umft.ro; 8 Department of Pulmonology, ‘Victor Babes’ University of Medicine and Pharmacy Timisoara, 300041 Timisoara, Romania

**Keywords:** CT-guided transthoracic biopsy, minimally invasive lung biopsy, core-needle biopsy lung, pulmonary nodule diagnosis, lung biopsy safety, CT-guided lung biopsy efficacy, lung cancer diagnosis, pneumothorax in lung biopsy, pulmonary hemorrhage biopsy, transthoracic biopsy complications

## Abstract

CT-guided transthoracic core-needle biopsy (CT-TTNB) is a minimally invasive procedure that plays a crucial role in diagnosing pulmonary nodules. With high diagnostic yield and low complication rates, CT-TTNB is favored over traditional surgical biopsies, providing accuracy in detecting both malignant and benign conditions. This literature review aims to present a comprehensive overview of CT-TTNB, focusing on its indications, procedural techniques, diagnostic yield, and safety considerations. Studies published between 2013 and 2024 were systematically reviewed from PubMed, Web of Science, Scopus, and Cochrane Library using the SANRA methodology. The results highlight that CT-TTNB has a diagnostic yield of 85–95% and sensitivity rates for detecting malignancies between 92 and 97%. Several factors, including nodule size, lesion depth, needle passes, and imaging techniques, influence diagnostic success. Complications such as pneumothorax and pulmonary hemorrhage were noted, with incidence rates varying from 12 to 45% for pneumothorax and 4 to 27% for hemorrhage. Preventative strategies and management algorithms are essential for minimizing and addressing these risks. In conclusion, CT-TTNB remains a reliable and effective method for diagnosing pulmonary nodules, particularly in peripheral lung lesions. Advancements such as PET/CT fusion imaging, AI-assisted biopsy planning, and robotic systems further enhance precision and safety. This review emphasizes the importance of careful patient selection and procedural planning to maximize outcomes while minimizing risks, ensuring that CT-TTNB continues to be an indispensable tool in pulmonary diagnostics.

## 1. Introduction

### 1.1. Background and Importance of CT-Guided Transthoracic Needle Biopsy

CT-guided transthoracic needle biopsy (CT-TTNB) is essential for diagnosing and managing pulmonary nodules. This minimally invasive procedure offers high diagnostic accuracy and low complication rates, making it a critical tool in clinical practice [1].

CT-TTNB has revolutionized lung pathology diagnosis, providing precise needle placement and high diagnostic yield, surpassing older, more invasive methods. With sensitivity and specificity rates often exceeding 90%, CT-guided TTNB ensures timely and accurate diagnoses, aiding in effective therapeutic decision-making [2].

Compared to surgical biopsies, CT-TTNB is less invasive, involves lower morbidity, costs, and recovery times, and can often be performed on an outpatient basis with local anesthesia [3].

The procedure aids in staging lung cancer, which is crucial for determining disease extent and planning treatment, by obtaining tissue samples from primary and metastatic sites [1].

Furthermore, the integration of CT-TTNB with lung ablation procedures allows for simultaneous diagnostic and therapeutic procedures. This combined strategy is especially beneficial in managing selected cases, where these combinations demonstrate efficient and comprehensive care for patients with lung tumors [4].

### 1.2. Objectives of the Review

This review aims to provide a thorough understanding of CT-TTNB, its role in diagnosing and managing pulmonary nodules, and considerations for maximizing its efficacy and safety in clinical practice.

It describes CT-TTNB indications, absolute and relative contraindications, and patient selection strategies to reduce risks and optimize outcomes.

The review covers patient positioning, needle selection, and imaging guidance step-by-step. It also examines biopsy needles and their use in CT-TTNB, data on diagnostic yield, sensitivity, specificity, and accuracy, and comparisons with other pulmonary nodule diagnostic procedures. The review tackles preventing and treating pneumothorax (PNX) and pulmonary hemorrhage to improve patient safety.

Furthermore, it includes a decision-making flowchart to guide clinicians through the indications, procedural steps, and post-procedural care for CT-TTNB, and discusses potential areas for research and development in the field of pulmonary diagnostics.

## 2. Materials and Methods

The selection of articles for this narrative review adhered to the guidelines of SANRA (Scale for the Assessment of Narrative Review Articles). The literature search was carried out in July 2024 utilizing the PubMed, Web of Science, Scopus, and Cochrane Library databases, focusing on studies published from 2013 to 2024, covering a 10-year span.

We utilized the following MeSH terms for the PubMed search: (“CT-guided biopsy” [MeSH Terms]) OR (“pulmonary nodules” [MeSH Terms]) OR (“core needle biopsy” [MeSH Terms]) OR (“pulmonary efficacy diagnostic” [MeSH Terms]). The search strategy on Web of Science focused on the title, abstract, and keyword fields using terms such as “CT-TTNB”, “pulmonary nodule diagnosis”, “biopsy complications”, and “diagnostic yield”. The same protocol was employed for searches on Scopus and Cochrane Library. The inclusion criteria for the review were as follows:

- Studies published in English;
- Research conducted on human subjects;
- Studies evaluating the diagnostic yield, efficacy, and safety of CT-guided core-needle biopsy for pulmonary nodules;
- Clinical trials, cohort studies, and comparative studies focusing on biopsy techniques.

The exclusion criteria included the following:

1. Studies not published in English;
2. Research on animals or non-human subjects;
3. Studies involving participants under 18 years of age;
4. Research that did not focus on CT-guided biopsy or utilized other diagnostic techniques like bronchoscopy;
5. Studies on diseases other than pulmonary nodules (e.g., pathologies unrelated to lung cancer or benign pulmonary nodules);
6. Case reports or editorials.

A systematic and methodical search strategy was employed to retrieve the relevant literature, ensuring comprehensive coverage of studies related to the efficacy and safety considerations of CT-TTNB. The identification and selection process was based on the SANRA flow diagram and checklist to ensure transparency and reproducibility of the review.

**Figure 1:**
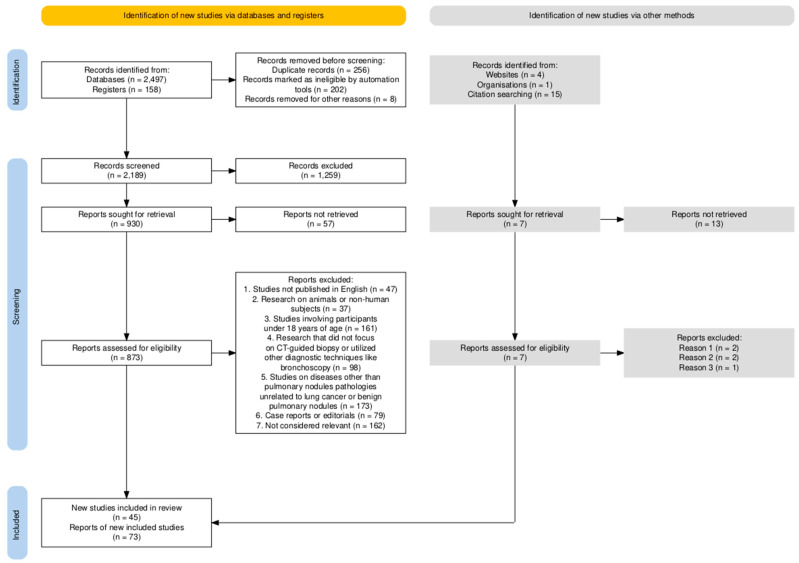
PRISMA flowchart. Literature review.

The diagram below illustrates the employed search strategy and the implemented filters. A step-by-step algorithm based on the SANRA statement is presented in Figure 1.

## 3. Indications

### 3.1. Evaluation of Solitary Pulmonary Nodules

Physicians aim to quickly identify a worrisome lesion and avoid unnecessary diagnostic or therapeutic treatments. Before biopsy, pulmonary lesions must be assessed for malignancy. Size, growth rate, density, shape, margins, internal characteristics, and complex findings are the key risk factors for pulmonary nodule malignancy [5].

The differential diagnosis for a solitary pulmonary nodule is complex and includes both benign and malignant causes. Early detection of lung tumors is essential, as the stage at which they are diagnosed has a critical role in determining the prognosis [6,7].

Nodule size and growth rate are frequently used criteria for evaluating the probability of nodule malignancy and establishing nodule management in line with international guidelines [8]. The management of a pulmonary nodule cannot depend just on its size, as there is a corresponding increase in the risk of malignancy as the diameter of the nodule increases. The incidence of cancer in nodules measuring less than 5 mm is extremely low, varying from 0 to 1% [9,10].

It is essential to perform morphological assessment utilizing thin-section computed tomography. A perifissural position, triangular form, interior fat, and benign calcifications are characteristics linked to benignity. Nodules that show spiculation, lobulation, pleural indentation, vascular convergence sign, associated cystic airspace, bubble-like lucencies, irregular air bronchogram, and sub-solid morphology are suggestive of malignancy [6,11].

The nodule’s density dictates its classification as solid or sub-solid. Solid nodules entirely conceal the underlying tissue, whereas sub-solid nodules exhibit a somewhat translucent appearance owing to the presence of ground glass. The density of ground glass exceeds that of normal lung tissue, while the conventional lung architecture is preserved, with appropriate bronchial and vascular borders. [12,13]. Sub-solid nodules can be further categorized as pure ground-glass nodules and part-solid nodules. The ground-glass component surrounding solid nodules is called a halo sign. It is important to identify sub-solid morphology because nodules with both solid and non-solid components have a higher probability of being cancerous [11,14,15].

Round or oval solid nodules have a lower likelihood of being malignant compared to more complex-shaped solid nodules. Conversely, sub-solid nodules with a round shape are more likely to be malignant [8]. Perifissural nodules, commonly intrapulmonary lymph nodes, are typically fissure-attached solid nodules with smooth margins and are often triangular or polygonal in shape. They can also appear oval or lentiform, usually within 15 mm of a pleural surface. Perifissural nodules with spiculated morphology or those crossing a fissure should not be classified as benign and require further investigation [6,16].

A smooth border is typically indicative of benignity. The presence of lobulation in a nodule is caused by varying or different rates of growth, which is strongly associated with the presence of malignancy. A lobulated border in part-solid nodules indicates invasiveness. Spiculation is a strong indicator of malignancy. It is caused by interlobular septal thickening and fibrosis, which happens when pulmonary vessels or lymphatic pathways become blocked by tumor cells [6,16,17].

Intranodular fat is characterized by a CT attenuation ranging from −40 to −120 Hounsfield Units. Fat presence is a sign of hamartoma. Calcification within a nodule usually corresponds to non-cancerous diseases. The form of calcification is vital for determining the probability of being benign or malignant. Benign calcifications are located in the center, they are typically linked to previous illnesses such as histoplasmosis and tuberculosis. Popcorn-shaped calcifications are a distinctive feature of chondroid calcifications in hamartomas. Malignant nodules generally have dystrophic calcifications that are more diffuse, shapeless, or punctate. These calcifications are usually limited in number and tend to be located near the periphery [6,18,19].

Cavitation in malignant solitary pulmonary nodules occurs due to necrosis of the central region and usually occurs in squamous cell carcinoma and metastasis. For differentiating benignity from malignancy, it is useful to evaluate the cavity morphology, irregularity, and wall thickness [6,17,19].

Complex findings, such as pleural retraction, air bronchogram in a setting of a solitary pulmonary nodule, bubble-like lucencies, and cystic airspace are also morphological features that suggest a malignant nature [6,17,19].

**Figure 2:**
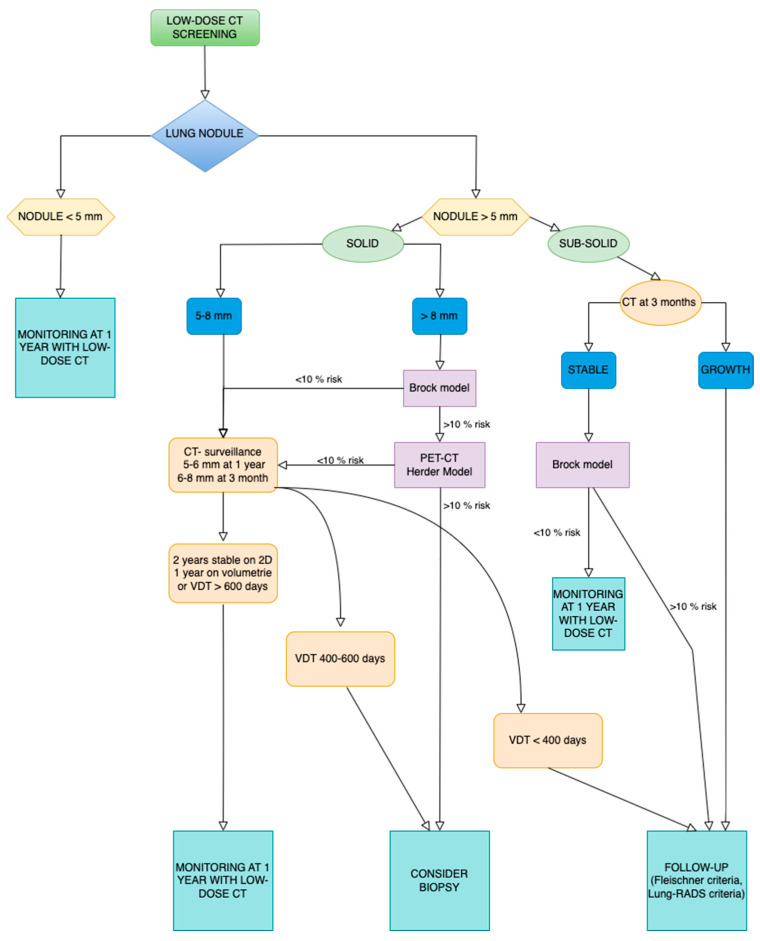
Protocol algorithm for establishing nodule management before CT-guided TTNB.

Figure 2 presents the protocol algorithm for establishing nodule management before CT-guided TTNB.

### 3.2. Metastatic Lung Disease

The indications for CT-TTNB in metastatic disease are to confirm when a lung nodule is suspected and to distinguish between primary lung cancer and metastatic lesions from other primary sites. Also, when imaging studies (CT, PET-CT) are inconclusive, and further tissue characterization is necessary to determine the nature of the nodule [20,21].

Another important indication is for therapeutic planning, to guide treatment decisions, including targeted therapies and immunotherapies, by obtaining tissue for molecular and genetic analysis, and to assess the response to treatment by comparing pre-treatment and post-treatment biopsy results [9,22].

The primary malignancy must be staged accurately, and this minimally invasive method can provide a tissue diagnosis. Prognosis and treatment depend on accurate staging. This minimally invasive procedure can also diagnose recurrence of previously treated lung cancer when new nodules appear, distinguish between infectious and malignant nodules, especially in immunocompromised patients where infections are a common differential, and obtain histological verification when non-invasive methods (sputum cytology, bronchoscopy) have failed [22,23].

## 4. Contraindications

### 4.1. Absolute Contraindications

The contraindications of percutaneous transthoracic needle biopsy can be classified into absolute and relative contraindications. Obtaining informed consent from the patient is required, and it involves providing a thorough description of the benefits and possible disadvantages of the procedure. CT-TTNB should not be performed if consent has not been obtained. CT-TTNB is absolutely contraindicated in cases of vascular lesions, such as pulmonary artery aneurysm or arteriovenous malformation, and in echinococcal cysts. Interventionists must carefully assess preoperative CT imaging to prevent the occurrence of severe hemoptysis during CT-TTNB as a result of vascular injury [5,23,24].

### 4.2. Relative Contraindications

Relative contraindications for TTNB include respiratory failure or predicted FEV1 of less than 35%, mechanical ventilation, bleeding tendency, coagulopathy, pulmonary arterial or venous hypertension, single lung (anatomically or functionally), severe interstitial lung disease, small lesions close to the diaphragm, central lesions, and uncooperative patients. Lung nodules situated juxtacardiac, juxtaphrenic, or in perivascular areas present special challenges when performing TTNB because of their proximity to vital anatomical structures. A thorough risk–benefit analysis must be considered when determining whether to proceed with the procedure. Although some medical centers have successfully biopsied central lesions, as shown by a study carried out by Mark C Murphy et al. [25], which involved biopsies of lesions adjacent to the heart followed by cryoablation, the associated risks demand careful evaluation. Lesions situated juxtaphrenic can provide additional challenges, such as increased chances of diaphragmatic injury and PNX. Specialized expertise and imaging assistance are necessary to reduce complications in these instances [26].

The minimal lung function required for percutaneous transthoracic needle biopsy is not specifically defined, however, it is usually accepted to have a predicted forced expiratory volume in one second (FEV1) of at least 35% as a threshold. A careful approach is crucial when doing CT-TTNB on patients requiring mechanical ventilation because these patients often exhibit increased lung motion caused by positive-pressure ventilation, which can reduce needle stability and increase the likelihood of complications such as PNX, air embolism, or hemorrhage. Strategies like utilizing advanced forms of ventilation like jet ventilation, which stabilizes the chest wall and reduces complications, or modifying ventilatory settings to minimize lung motion, should be taken into consideration if the procedure is considered necessary [27].

Prior to the procedure, it is important to rectify any bleeding tendencies and coagulation abnormalities. Pulmonary artery or venous hypertension is commonly acknowledged as a predisposing factor for post-procedure hemorrhage following CT-TTNB. However, the precise cutoff value for pulmonary arterial pressure as a contraindication for CT-TTNB remains undefined. Right-sided cardiac catheterization is the main method for diagnosing pulmonary hypertension by measuring the average pressure in the pulmonary artery. This technique is rarely conducted prior to CT-TTNB in clinical settings. If the mean pulmonary artery pressure is not available, an increase of the diameter of the main pulmonary artery to 2.95 cm or larger on a pre-procedural CT may indicate pulmonary hypertension [5,23,24].

Although it has been believed that a history of pneumonectomy is an absolute contraindication to CT-TTNB, Cronin et al. [28] reported that in patients with a history of pneumonectomy, the success rate of CT-TTNB, preferentially carried out by needle aspiration, was 86% (12 of 14) in a single lung. The decision to perform CT-TTNB must involve careful evaluation of the lesion’s features, the patient’s general health condition, and interventional competence in order to ensure patient safety and diagnostic effectiveness. Important is that the safety and success of CT-TTNB require patient cooperation. More specifically, throughout the entire process, the patient needs to remain in a stable position and adhere to breathing directions [23].

## 5. Technique

### 5.1. Patient Selection and Pre-Procedural Evaluation

A recent CT or PET/CT scan is needed to identify the lesion and determine the right patient. CT scans can help determine the best procedure for a pulmonary abnormality biopsy, such as a bronchoscopic biopsy, surgical biopsy (typically performed by video-assisted thoracoscopic surgery), or percutaneous lung biopsy. Bronchoscopy is recommended for central endobronchial and peribronchial lesions. Surgical biopsy is used to explore suspected interstitial lung disease or tiny peripheral lesions, especially those at the lung bases that a percutaneous needle biopsy cannot probe [3,21,29].

**Figure 3:**
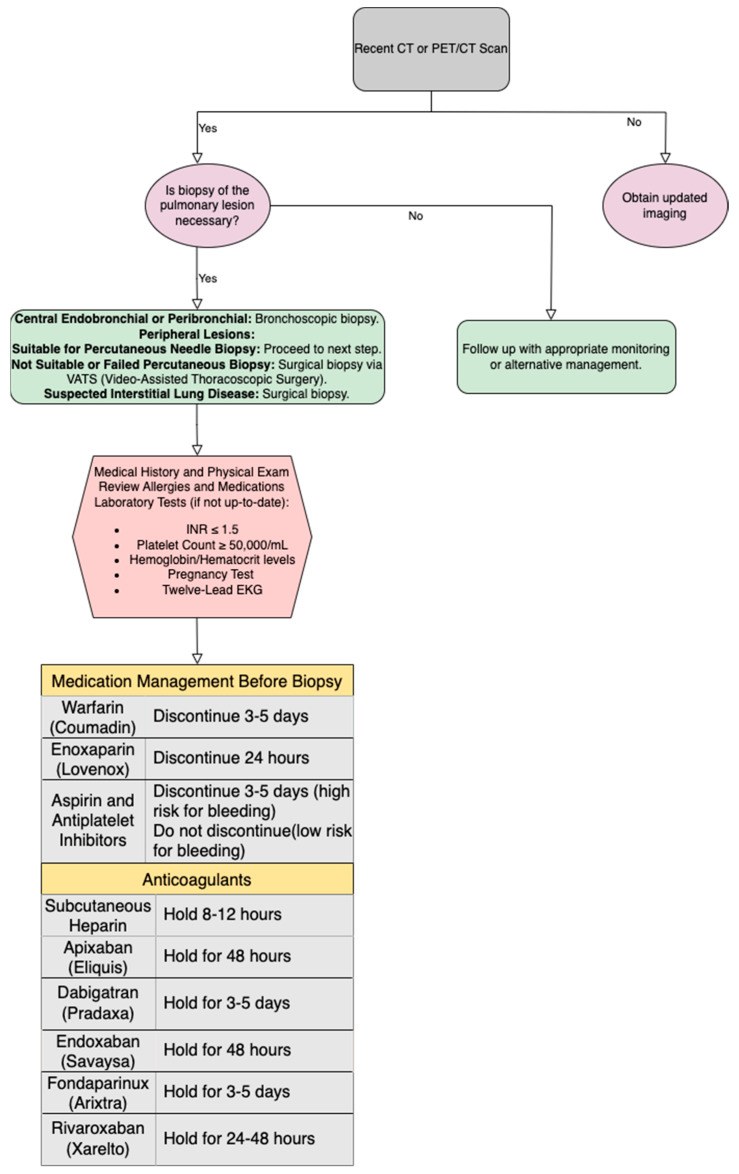
Protocol algorithm for pre-procedural evaluation and medication management before CT- TTNB.

If a percutaneous biopsy is considered to be needed and possible, the patient is sent to interventional radiology. Most interventional radiology practices typically examine their patients in a clinical setting before performing a biopsy. A comprehensive evaluation of the patient’s medical history and a thorough physical examination are conducted [29,30]. Figure 3 presents the protocol algorithm for pre-procedural evaluation and medication management before CT-TTNB.

Conducting a pre-procedural pulmonary function test and a multidisciplinary discussion in patients suspected of having severe chronic obstructive pulmonary disease (COPD) is essential.

Utilizing an 18F-fluoro-deoxyglucose PET scan to identify the precise location for a biopsy of a pulmonary lesion suspected to exhibit necrosis is important. PET imaging can identify the metabolically active part of a pulmonary lesion, which is likely to provide a conclusive outcome and the diagnostic accuracy of CT-TTNB is enhanced [29,31].

Contrast enhancement and high-resolution chest CT images aid biopsy planning. Contrast-enhanced CT images show cystic or necrotic areas in pulmonary lesions more clearly and can assist in the diagnosis of pulmonary vascular malformations. The interventionist can also detect hypertrophied systemic or pulmonary vessels to find the lesion and prevent vascular damage during the surgery [29].

Considering CT-based guidance modalities or multi-planar reconstruction for pulmonary lesions measuring 2 cm or smaller is crucial in order to enhance the diagnostic accuracy of CT-TTNB [23].

### 5.2. Patient Positioning

The prone position is better for patient placement than supine or decubitus. First, the back ribs move less than the front. Unlike the anterior area, the posterior ribs rotate during respiration but remain stable in the craniocaudal plane. During each inhalation, the chest expands and the ribs rotate, increasing the chest’s anteroposterior diameter while the front part moves craniocaudally. Since the ribs are not moving, a posterior approach reduces needle motion. The patient should be positioned with the biopsy site down after the procedure, another benefit of prone placement. After a procedure, patients prefer lying on their backs over being prone. Patients cannot observe the needle insertion when lying face down, another benefit of the posterior approach. This rarely bothers patients, although it can be very upsetting [32].

The lesion’s location and size, as well as the patient’s tolerance, should decide patient placement. Patient posture is critical for lung biopsy precision and safety. If a pulmonary nodule can be reached from prone, supine, or decubitus, prone is better and provides many benefits. To increase intercostal gaps for easier access, arms can be raised above the head [30].

To minimize the risk of PNX, the biopsy needle does not need to cross the interlobar fissure. Oblique and decubitus positions are less stable than supine or prone positions, however they can be used to access a subpleural lesion on the side of the lungs without damaging normal lung tissue [29]. 

The pathway for access is determined based on CT imaging. When selecting an access route, it is crucial to avoid chest wall vessels such as the subclavian, internal mammary, intercostal, and intrapulmonary vessels. It is crucial to reduce pleural transgression by only performing one puncture in the pleura and avoiding any fissures if feasible. Avoiding large bullae is also recommended. If a biopsy is being conducted for a lesion with mixed CT attenuation, it is advisable to target the solid component of the nodule or mass. Likewise, if there is a presence of central necrosis, it is preferable to perform a biopsy specifically targeting the wall of the lesion [33].

### 5.3. Sedation and Anesthesia

Sedation during CT-TTNB is rarely advised because it reduces patient participation. Sedated patients have trouble following instructions and are more likely to experience unexpected movements during the treatment. Rare cases of intense anxiety or claustrophobia may be exempt. Oral alprazolam 0.25 mg or intravenous midazolam 1 mg can be given for sedation [32]. 

The administration of local anesthetic is performed with the guidance of CT imaging along the planned biopsy path using 1% lidocaine solution. Local anesthesia is administered in all layers of the chest wall, up to the level of the parietal pleura, but without crossing it (crossing it increases the risk of PNX). When the needle approaches the lower edge of the rib cage, lidocaine should be given to manage any pain that may occur from the nerves between the ribs. The patient should be informed that sensations caused by lidocaine may spread throughout the entire rib [34].

### 5.4. Biopsy Needles and Equipment

#### 5.4.1. Types of Needles (Aspiration, Cutting)

Percutaneous biopsies use fine-needle aspiration and cutting or core needles. Fine-needle aspiration biopsy is cytology. Cytological and microbiological samples are aspirated with 20–25-gauge needles. Core or cutting biopsy needles offer tiny tissue samples for histology. The needles are usually larger than aspiration needles. Small diameter (18–20 gauge) automated cutting needles are also available [35].

These needles can be built either as end-cutting or side-cutting devices. The Tru-Cut is the predominant core biopsy needle, including an exterior cutting cannula and an inner slotted stylet [31].

#### 5.4.2. Selection Criteria for Needles

The selection of a needle is determined by factors such as the size and position of the lesion, the preferred route that the needle will take, the information that is expected to be obtained from the sample, the status of the lung tissue, and the personal preference of the operator [34].

A single needle or coaxial approach can be used for aspiration and core biopsies. Single-needle surgery involves inserting a needle into the lesion. Repeated passes are made if many samples are needed. Alternatively, a coaxial technique involves placing an outer guiding needle near the target and threading a narrower biopsy needle through it to sample the lesion. The single-needle technique requires imaging guidance every time the needle is placed into the lesion, which prolongs the procedure. Each procedure crosses intermediate structures, increasing the risk of problems. A coaxial technique has several benefits, but it requires larger guiding needles to penetrate the pleura. Using a coaxial approach with a lesion with a prominent internal air-bronchogram or open-bronchus sign increases the chance of an air embolism. Lesion depth from the skin should determine the needle length [36].

There are two specimen notch lengths: 10 mm and 20 mm for a core needle biopsy. A longer specimen notch is favored due to its ability to provide a larger amount of tissue. However, in cases when the lesion is tiny, a shorter notch is utilized to prevent damage to the normal tissues. After careful examination of the prior images, a path that ensures safety is selected to reach the lesion, along with a needle of proper size [33].

Core biopsy alone or core biopsy and fine-needle aspiration (FNA) depends on many parameters including the institution and operator. It is unclear how many passes each procedure requires. The complexity of the technique, the danger of complications, the quality of the initial specimen, and the pathologist’s requests determine whether to perform multiple punctures. An on-site pathologist may reduce biopsies [33,37].

### 5.5. Procedural Steps

#### 5.5.1. Planning the Access Route

It is advisable to position the needle access site in the cephalic region adjacent to the ribs in order to prevent accidental puncture of intercostal vessels and nerves. When using the anterior parasternal way, it is important to be careful and avoid the internal mammary blood vessels. If necessary, the costal cartilage can be traversed, but doing so may decrease the mobility of the needle [37].

In general, lung biopsies follow the shortest intrapulmonary path, avoiding fissures, bullous lesions, and emphysematous areas. The path is determined by identifying the shortest possible distance through the lung parenchyma from the needle entry point to the lesion. The calculation eliminates intercostal structures and concentrates exclusively on lung tissue. The length is measured from the pleural surface (entry point) to the center or periphery of the lesion, depending on the biopsy target. Collapsed or consolidated lung tissue may occur with lung masses. Intravenous contrast is better in this circumstance since a mass displays less enhancement than collapsed lung tissue and vascular lines are not visible [35].

To position the table for biopsy plane marking, the best axial image is found. The patient is relocated in the CT gantry while preserving the table position. Laser light from the scanner marks this place on the patient’s skin. The grid superimposition method determines the lesion depth and the distance between the CT gantry midline and the predicted skin entrance point. After cleaning the area, iodine-based contrast media or hypodermic needle dots are placed on the skin mark. For confirmation, a scan is performed at that level. A measuring scale and laser light aligned with the midline are used to determine the laser application site on the skin. An inedible marker is used to indicate the skin entry. If an inedible marker is unavailable, the needle hub back can be used to make a skin impression. Following that, a local anesthetic is administered and, if necessary, a minor cut is made on the skin using a no. 11 scalpel [38].

#### 5.5.2. Needle Insertion and Positioning

The guiding needle is inserted into the soft tissue of the chest wall. It is important to make all required adjustments to ensure that the needle is exactly aligned with the target lesion before penetrating the pleura. All needle manipulations, including those in the chest wall, must be carried out while the patient is in the specified breath-hold position. The CT console can show the needle’s anticipated trajectory by extending it toward the lesion, or the needle tip’s beam hardening artifact can show it. Afterwards, the needle is inserted in a single motion into the lung until it reaches the edge of the lesion in cases where the lesion is located peripherally, or at least 1–2 cm inside the lung parenchyma, measured from the pleural surface. To prevent the needle from slipping into the pleural space during respiratory motion and to avoid laceration of the pleura, it is advisable to leave a needle inside the lung parenchyma, with a distance of at least 1–2 cm, especially in the lower lung regions. It has been recommended that the minimum depth for needle insertion should be 1.5 cm for the lower lobes and 1.0 cm for the upper lobes. Once the needle has entered the lung, it should be allowed to move freely with each breath and should only be touched during the specified breath-hold [35].

Short needle lengths in the lung can readily become dislodged during respiratory motion and can rip the pleural surface, making taking a biopsy of tiny subpleural lesions difficult. Some writers sample subpleural lesions tangentially instead of at a straight angle. This is because the tangential path improves needle stability and correction [39].

While studies indicate that there is no connection between the number of passes performed and the occurrence of PNX, other authors have discovered that multiple punctures have been associated with higher risks of PNX and procedure failure. In order to reduce complications, it is advisable to puncture the pleura only once. A minor to moderate PNX developing during the procedure does not require stopping of the procedure, as long as the PNX remains stable. However, a postoperative PNX may progress to moderate or severe forms, requiring close monitoring [32].

The needle should be progressively advanced from the skin towards the lesion. It is important to continuously monitor the most recent scan to ensure precise planning, as there are multiple factors that can cause the lesion to move during the procedure, such as patient movement, cardiac and respiratory motion, and the development of PNX. Respiratory motion must be carefully considered during the procedure. The lower lobes typically demonstrate increased respiratory excursion compared to the upper lobes. Certain operators choose to permit patients to breathe freely during the procedure. Patients may also be advised to breathe normally, minimizing large respiratory movements. In contrast, some individuals opt for breath-holding techniques during imaging and needle positioning to maintain steadiness with the lung anatomy and the needle movement. For stiff needles, like bigger gauge needles or coaxial system guiding needles, the needle is partially retracted and the tip is redirected by pressing the hub in the appropriate direction while applying skin pressure. Keep the needle at the right angle as it moves forward toward the lesion. Do not remove and reposition the needle tip through the pleura while manipulating the lung. Adjusting the targeted breath-hold may align the lesion with the needle path to correct the craniocaudal needle tip misalignment in the lung. If the lesion is above the needle tip, partially withdrawing the needle from the lung’s periphery, deep breathing, and pressing the needle forward may fix it [35].

#### 5.5.3. Sample Collection and Handling

Proper handling of the collected tissue is essential to maintain its integrity and diagnostic value. Once the tissue sample is obtained, it must be promptly and carefully removed from the needle and placed in an appropriate medium, typically formalin, to preserve cellular architecture. Special attention is given to the sample’s orientation, especially if multiple cores are taken, to ensure that pathological evaluation can accurately reflect the nodule’s structure and composition [40].

Biopsy samples must be transported to the pathology lab according to strict protocols to avoid degradation or contamination. If delays occur, samples should be transferred in sealed, labeled containers in a chilled environment. Pathology labs should be notified of incoming samples to ensure prompt processing. Delays in molecular or genetic testing can dramatically affect outcomes [41].

The macroscopic evaluation of tissue samples obtained from CT-TTNB of pulmonary nodules is a crucial first step in the diagnostic process. Upon collection, the tissue sample is typically examined visually to assess its overall appearance. The macroscopic characteristics that are often noted include the size, shape, color, and consistency of the tissue. These aspects can provide initial clues about the nature of the nodule, although definitive diagnosis requires microscopic analysis [40].

Macroscopic features analyzed in the biopsy sample include size and form. Core biopsy specimens are cylindrical and vary in length and diameter based on the needle gauge and procedure. To provide enough material for histological investigation, a core should be 1–2 cm long and 1–2 mm wide. An adequate sample size reduces the risk of a non-diagnostic result and allows for thorough examination, including molecular testing. Color of the tissue sample can also indicate pulmonary nodule kind. Malignant tissues are sometimes more heterogeneous, with necrosis or bleeding giving them a tan to reddish-brown color. Benign lesions are light or colorless. Necrotic tissue or hemorrhagic patches can suggest aggressive or severe disease and can help the pathologist pick locations for further study. Consistency and texture are also assessed in biopsy samples. Unlike benign or inflammatory lesions, malignant tissues may feel harder or fibrous. Fibrotic or sclerotic alterations may be more common in chronic inflammatory diseases or desmoplastic reactions associated with various malignancies [41].

The macroscopic examination is essential for identifying any immediate issues with the biopsy sample that might necessitate additional sampling. For example, if the initial sample appears fragmented or is deemed insufficient in size, an additional pass with the needle might be performed to obtain more tissue. Similarly, any signs of crush artifacts or other forms of damage can alert the clinician to potential problems that could compromise the diagnostic yield, prompting a review of technique or handling procedures [41].

In summary, the macroscopic assessment of pulmonary biopsy samples involves evaluating the size, shape, color, and consistency of the tissue. These initial observations can provide valuable insights and ensure that the sample is adequate for further histopathological examination, thereby playing a pivotal role in the accurate diagnosis and management of pulmonary nodules [42].

#### 5.5.4. Patient Monitoring

The implementation of multidisciplinary management strategies for lung lesions may substantially improve diagnostic and therapeutic decision-making. This methodology combines pulmonology, thoracic surgery, radiology, and interventional teams to provide comprehensive patient care [43].

Post-procedural care is a critical component in ensuring the safety and well-being of patients undergoing CT-TTNB of pulmonary nodules. This phase involves careful monitoring and immediate imaging to detect and manage potential complications early.

After a CT-TTNB biopsy, patient monitoring is essential to detect immediate or delayed consequences. The main patient monitoring aspects:

- Monitoring vital indicators like heart rate, blood pressure, respiratory rate, and oxygen saturation is essential. This helps detect PNX and bleeding early.
- Monitoring respiratory status is crucial due to the process. Dyspnea, hemoptysis, and diminished breath sounds may suggest a PNX or associated problems.
- Regular pain assessment using standard scales is necessary for effective pain management. Analgesics should be given to relieve procedure pain and guarantee patient comfort.
- Regularly check for problems such as PNX, pulmonary hemorrhage, and infection. Physical examination and patient-reported symptoms [32].

#### 5.5.5. Immediate Post-Procedure Imaging

Post-procedure imaging is essential for evaluating biopsy success and detecting early complications. Computed Tomography is now recognized as the predominant imaging technique due to its exceptional diagnostic ability, particularly in identifying early complications such PNX, pleural effusion, and hemorrhage. However, the early detection of air embolism is the most critical aspect, as this complication, though rare, is directly associated with mortality if not promptly identified and treated [44,45].

A CT scan performed immediately post-procedure is very effective in detecting the two predominant post-biopsy complications: PNX and hemorrhage. The CT obtains comprehensive images of the biopsy site immediately post-needle removal, facilitating the early identification of minor pneumothoraxes, hemorrhages, or the presence of an air embolism that may be overlooked by alternative imaging methods. CT detected 80.5% of PNX instances that were later detected on chest radiographs, while it has a 6% false-negative rate, resulting in some PNX cases being overlooked but identified on follow-up imaging. Moreover, bleeding identified around the puncture site during CT has been demonstrated to diminish the incidence of PNX, presumably due to clot formation that aids in sealing the pleura [44]. Identifying an air embolism during CT-guided transthoracic needle biopsy is crucial, as even minimal air volumes in critical circulations, such as the left atrium, coronary arteries, or cerebral arteries, can result in life-threatening consequences, including stroke, myocardial infarction, or cardiac arrest. Emboli often appear rapidly with symptoms like hypotension, loss of consciousness, or hemiplegia, requiring urgent interventions [45].

Post-procedure, patients are observed for 2 h after surgery, and any clinical decrease requires additional imaging and intervention. If no deterioration is seen, a follow-up chest X-ray is performed to confirm and catch any abnormalities. Due to its cost, chest radiographs are still used in some situations even though CT is better. To diagnose PNX, it is usually performed within an hour of biopsy. Chest radiographs are less sensitive than CT and often miss small or occult pneumothoraxes. For patients with negative CT results, a follow-up chest X-ray is necessary [46].

Ultrasound is sometimes utilized post-procedure, mainly to detect pleural effusions or hematomas. However, its use in detecting PNX is limited, making it less preferred compared to CT or chest radiograph. Ultrasound is most useful for evaluating localized fluid collections near the pleura [47].

## 6. Efficacy

### 6.1. Diagnostic Yield and Accuracy

The diagnostic yield of CT-TTNB refers to the percentage of biopsies that result in a definitive diagnosis. Studies report a high diagnostic yield for CT-TTNB, typically ranging from 85% to 95%. This variation is influenced by factors such as nodule size, location, and the experience of the operator. For instance, a study by Choi et al., which included 1216 patients, found that the diagnostic yield was significantly higher for nodules larger than 2 cm in diameter, with a yield of 95%, compared to 82% for smaller nodules. Similarly, the yield is higher for peripheral nodules compared to centrally located ones [32,48,49].

Diagnostic accuracy is a measure of how correctly the biopsy identifies the presence or absence of malignancy, typically evaluated through sensitivity, specificity, and positive predictive value. The sensitivity of CT-TTNB for detecting malignant pulmonary nodules is reported to be between 92% and 97%, with a specificity of 85% to 95%. These values indicate that the procedure is highly reliable in confirming malignancy when present and fairly accurate in ruling it out when absent [50,51].

A comprehensive meta-analysis by Wu et al. reviewed 30 studies encompassing over 5000 patients, revealing a pooled sensitivity of 94% and a specificity of 89%. The study highlighted that while CT-TTNB is highly accurate for diagnosing malignancies, its accuracy in diagnosing benign lesions is somewhat lower, often necessitating further diagnostic procedures [1].

Larger nodules and those with spiculated edges are more likely to yield accurate results. Experienced radiologists and pathologists contribute to higher accuracy and yield. The use of coaxial techniques and rapid on-site evaluation (ROSE) can enhance diagnostic accuracy by ensuring adequate sample quality [52,53].

CT-TTNB has biases and limits despite its great diagnostic yield and accuracy. Studies that choose patients with larger, easier-to-biopsy nodules may have selection bias. Only positive biopsy results followed up with surgical confirmation may influence sensitivity and specificity estimates. Operator-dependent variability can also cause yield and accuracy discrepancies among studies and clinical situations. These factors should be considered when interpreting the findings and applying them to broader patient populations [33,54].

### 6.2. Sensitivity and Specificity for Malignancy

Sensitivity measures the ability of the biopsy to correctly identify malignant nodules, while specificity reflects its ability to correctly identify non-malignant cases [55,56].

CT-TTNB is known for its high sensitivity in detecting malignant pulmonary nodules. Studies report sensitivity rates typically ranging from 92% to 97%. A meta-analysis by Heerink et al. (2017), which included 48 studies with a total of 10,865 patients, found a pooled sensitivity of 94.5% for CT-TTNB in diagnosing lung cancer [57].

The experience of the radiologist performing the biopsy and the use of advanced imaging techniques, such as CT fluoroscopy, can further enhance the sensitivity by improving needle placement accuracy [49,58].

However, specificity can be affected by various factors. For example, false positives can occur if benign conditions such as granulomas or infections are misinterpreted as malignancies based on biopsy samples. In cases where benign nodules exhibit atypical or suspicious features on imaging, the specificity might be lower, leading to potential overdiagnosis [32,51].

When compared to other diagnostic modalities, CT-TTNB offers a favorable balance of sensitivity and specificity. Fine-needle aspiration (FNA), another commonly used method, tends to have a higher specificity but slightly lower sensitivity compared to CT-TTNB. Conversely, CT-TTNB’s ability to obtain larger tissue samples provides a more definitive histopathological diagnosis, especially in differentiating between various types of malignancies or benign conditions [55,56,59,60].

### 6.3. Factors Influencing Diagnostic Success

#### 6.3.1. Lesion Size

The size of the pulmonary nodule is a significant determinant of diagnostic yield in CT-TTNB. Larger lesions generally provide a higher diagnostic accuracy due to the greater volume of tissue available for sampling. In other studies, while lesions measuring 41–50 mm had a high diagnostic accuracy of 95%, this rate decreased to 80% for lesions over 50 mm. This result contrasts with earlier work, which reported a diagnostic rate of 100% for lesions exceeding 50 mm. One possible explanation for this discrepancy could be the central necrosis in larger lesions. Additionally, larger lesions may be associated with collapse and consolidation of the surrounding lung tissue, complicating the biopsy process and contributing to inaccurate sampling [7,48,61].

Conversely, smaller lesions, particularly those less than 10 mm, present a greater challenge, often resulting in lower diagnostic yields due to the difficulty in accurately targeting and sampling sufficient tissue [62,63,64].

Other studies have examined the effect of lesion size on diagnostic accuracy by dividing patients into groups based on specific cutoff points. For example, Laurent et al. [65] used 20 mm as a threshold, while Li et al. [53] opted for 15 mm. Lesions measuring 15 mm or less had a diagnostic rate of 60%, compared to 82% for lesions larger than 15 mm. The reduced diagnostic success in smaller lesions is likely due to an increased risk of sampling error, as smaller lesions present a more challenging target for precise needle placement during biopsy [53].

#### 6.3.2. Lesion Depth

Since the axial plane of CT imaging serves as the standard imaging window for biopsy procedures, it serves as the basis for the classification of central and peripheral lung lesions. Central lesions are located in the inner one-third of the hilar costal diameter, corresponding to regions near the hilum, main bronchi, and central vasculature. Middle lesions are located in the middle one-third of the hilar costal diameter, serving as a transitional zone between central and peripheral regions. Peripheral lesions are found in the outer one-third of the hilar costal diameter, including pleural-attached nodules, which are nodules abutting or directly attached to the pleura [66]. In a comprehensive series by Yeow et al. [67] involving 660 percutaneous transthoracic lung biopsies (PTLBs), lesion depth emerged as the most significant predictor of PNX. The study found that lesions abutting the pleural surface had the lowest risk of PNX, while subpleural lesions located between 1 mm and 20 mm in depth posed the highest risk. Interestingly, the risk of PNX decreased as lesion depth increased beyond this range. To mitigate this risk, the authors suggested using a longer, oblique needle path to prevent inadvertent dislodgement of the guiding needle, an approach also supported by other researchers [67].

Similarly, Khan et al. [68] found that the PNX rate was significantly higher when lesions were located within the lung parenchyma compared to pleural lesions (*p* < 0.05).

However, all PNX instances needing chest tube implantation occurred in lesions less than 2 cm from the pleura, underscoring the role of lesion proximity in predicting more severe consequences [69].

Additionally, fine-needle aspiration (FNA) techniques have demonstrated that increased lung parenchyma traversed during the biopsy procedure elevates the risk of complications, including PNX. These findings underscore the complex relationship between lesion depth and diagnostic success, with shallower lesions near the pleural surface posing unique risks that require careful management during CT-TTNB procedures [70].

#### 6.3.3. Number of Biopsy Passes

In CT-TTNB of pulmonary nodules, the number of biopsy passes is a pivotal factor influencing the diagnostic success rate [71,72].

Multiple biopsy passes improve diagnostic yield, especially in getting enough tissue for histopathological and genetic testing. In precision medicine, genetic markers dictate lung cancer treatments, making molecular testing essential. Submitting tissue samples in several cassettes, typically needing multiple biopsy passes, considerably enhances molecular testing tissue adequacy, according to a 2021 study. The same study found that more than two core samples are often needed, especially for sophisticated molecular analysis to inform therapy options [2].

Another study from 2022 highlighted that when fewer than two passes were performed, the likelihood of diagnostic failure increased significantly, especially for smaller or more challenging lesions. This suggests that while the number of passes should be minimized to reduce patient discomfort and risk, ensuring enough passes are made to obtain a representative tissue sample is essential [32].

The risk of complications, such as PNX, hemorrhage, or even air embolism, tends to increase with the number of biopsy passes. Each additional pass increases the likelihood of puncturing lung tissue or blood vessels, which can lead to serious complications. For instance, a recent study found that the incidence of PNX was significantly higher in cases where more than three passes were performed [3,72].

Moreover, the use of advanced imaging techniques, such as PET/CT fusion imaging, can help guide the biopsy needle more accurately, potentially reducing the number of necessary passes while still achieving high diagnostic accuracy. This approach can help target the most metabolically active regions of a nodule, which are more likely to yield diagnostic tissue, thus improving the overall success rate of the procedure [3,21,73].

#### 6.3.4. Needle Path

In CT-TTNB of pulmonary nodules, the length of the needle path from the pleura to the target lesion is a critical factor that can significantly influence diagnostic success. The needle path is typically measured as the distance from the pleura to the target lesion, traversing lung parenchyma. This measurement is made on axial CT images during procedural planning, ensuring an accurate representation of the distance the needle must travel. The needle path length impacts both the accuracy of the biopsy and the risk of complications, making it an essential consideration in planning and performing the procedure [32,67,72].

Needle path length is directly related to the complexity of the biopsy procedure. A shorter needle path, typically found in lesions closer to the pleural surface, tends to facilitate easier access, resulting in higher diagnostic accuracy [59]. This is because the shorter path minimizes the distance that the needle must travel through the lung parenchyma, reducing the risk of needle deflection and improving the precision of targeting the nodule. A 2022 study emphasized that shorter needle paths are associated with improved diagnostic yield and lower rates of nondiagnostic results, particularly in smaller pulmonary nodules [32].

Conversely, a longer needle path, often required for deeper or centrally located lesions, presents more challenges. Research has shown that lesions requiring longer needle paths are associated with a higher incidence of complications, which can compromise the overall effectiveness of the biopsy [2,50].

#### 6.3.5. Imaging Guidance and Techniques

The precision of the biopsy, the quality of the tissue samples obtained, and the minimization of complications are all significantly influenced by the imaging modalities and techniques employed during the procedure [51].

Recent advancements in imaging technology have substantially improved the accuracy and safety of CT-TTNB. One notable development is the integration of PET/CT fusion imaging, which combines functional and anatomical data to better guide needle placement. A 2022 study demonstrated that PET/CT fusion imaging significantly increases diagnostic yield, particularly in complex cases where standard CT imaging might not adequately distinguish between benign and malignant regions within a nodule. This technique allows for more precise targeting of metabolically active areas of a lesion, which are more likely to provide diagnostic tissue [3,73].

Real-time imaging, such as continuous CT fluoroscopy, is another critical factor influencing the success of CT-TTNB. This technique allows for continuous visualization of the needle as it approaches and penetrates the target lesion, enabling the radiologist to make real-time adjustments. According to a study published in Frontiers in Medicine in 2022, real-time needle tracking during the biopsy significantly reduces the risk of needle misplacement, which in turn decreases the likelihood of complications and increases the chances of obtaining a diagnostic sample. The quality of the imaging used during CT-TTNB also plays a vital role. Studies have shown that using thin-slice CT imaging, typically with a slice thickness of 2.5 mm or less, enhances the resolution of the images, allowing for better visualization of small nodules and more accurate needle placement. The same study in *Frontiers in Medicine* noted that thin-slice imaging, combined with advanced image reconstruction techniques, improves the ability to identify and target small or faint lesions, thereby increasing the diagnostic yield and reducing the need for repeat procedures [2,58].

Cone-beam computed tomography (CBCT) has become an effective imaging technique for facilitating transthoracic core-needle biopsy (CNB) of lung lesions. Its capacity to deliver real-time, high-resolution, three-dimensional imaging during procedures has considerable benefits compared to traditional computed tomography (CT), especially regarding the accuracy of needle insertion and the management of small or challenging lung lesions. CBCT offers real-time 3D imaging, enabling continuous guidance during the biopsy procedure. This real-time imaging increases needle targeting by allowing the operator to dynamically modify the needle’s direction according to the lesion’s position during respiration. Conversely, traditional CT offers a more static imaging methodology, necessitating periodic scans and repositioning for needle guidance. CBCT integrates virtual guidance software that improves the biopsy procedure. Through the integration of 3D imaging and virtual guidance, the operator can ascertain the best needle trajectory before insertion and obtain feedback regarding the needle’s position in relation to the target during the procedure. This real-time feedback reduces the number of needle repositions, minimizing tissue trauma, radiation exposure, and procedural time [74].

The diagnostic accuracy of CBCT for lung nodule biopsy is well-documented. In studies focusing on juxtaphrenic lesions, for example, CBCT-guided CNB demonstrated a diagnostic accuracy of 92.7% with a high positive predictive value (96.9%) for detecting malignancy. The ability to consistently visualize the needle and lesion during real-time imaging has made CBCT particularly effective in targeting small or deep-seated nodules that are challenging to access with conventional CT guidance [26].

While both CBCT and conventional CT are associated with risks such as PNX and hemoptysis, studies have shown that CBCT-guided procedures have comparable or slightly reduced complication rates. In a comprehensive multicenter analysis, the incidence of PNX and hemoptysis was comparable between CBCT and conventional CT; however, CBCT was linked to reduced radiation exposure and fewer needle repositioning maneuvers. In patients with juxtaphrenic lesions, a particularly challenging biopsy site, CBCT decreased the overall complication rate while maintaining an excellent diagnostic yield [72].

The future of image-guided biopsies probably depends on the continued enhancement of CBCT technology and its incorporation with sophisticated navigation systems to enhance diagnostic precision and patient safety [75].

Sensitivity and specificity comparison between cone-beam CT and conventional CT are summarized in Table 1.

CBCT offers slightly lower complication rates than CCT, especially in terms of PNX, hemorrhage, and chest tube insertion, due to its real-time guidance and fewer needle repositioning needs. Additionally, CBCT significantly reduces radiation exposure, making it a safer choice in terms of patient safety, particularly in more complex or high-risk biopsies. However, CCT is still highly reliable and remains widely used due to its availability and faster procedural time in straightforward cases [76,77].

Complications comparison between cone-beam CT and conventional CT are summarized in Table 2.

## 7. Complications

### 7.1. Pneumothorax

#### 7.1.1. Incidence and Risk Factors

PNX is the most frequent complication that arises during or soon after a PTLB; it is known to have an estimated occurrence rate of 12–45%, resulting in a chest tube installation rate of 2–15% [72,78],.

Several factors have been associated with a higher risk of PNX, including the size and depth of the lesion, the presence of emphysema, the location in the lower lobes, the number of pleural punctures, the type of needle used, and the level of training of the radiologist performing the procedure [47,67,70].

Patients with lung lesions of less than 2 cm have a PNX rate reaching 33%. Across this subset of patients, there is no noticeable difference in the occurrence of PNX across radiologists with different levels of experience. In contrast to the other radiologists, who had an average PNX rate of 29%, the pioneer radiologist had a 17% rate for lesions > 2 cm. Lesion depth is the second risk factor for PNX. The incidence of PNX increases significantly from 13% when lesions are adjacent to the pleural surface to 29% when aerated lung is traversed. Biopsies on tiny lesions are tough because they take longer and require more needle modifications, and it may increase complications [62,64,67,79].

Yeow et al. [67] found that there was a notable rise in the occurrence of PNX, from 13% for lesions adjacent to the pleural surface to 29% for lesions when the needle passes into aerated lung. This increase can be explained by the reduced stability of the needle as it travels a shorter path within the lung, leading to breaches in the pleura. A study showed that using a longer transpulmonary path resulted in a decreased incidence of PNX and an increased rate of successful biopsy for subpleural lesions [80]. Some claim that a needle pathway longer than 4 cm is linked to a greater occurrence of PNX [81].

The experience of the radiologist is the third significant risk factor for PNX, with a 17% rate of PNX for biopsies conducted by experienced radiologists compared to 30% for other radiologists [67].

Chuang He et al. [82] discovered no significant difference in the occurrence of PNX when performing the procedure in the needle size range of 16 to 19 G. These findings contrast with those of the retrospective study, which reported that inserting a smaller needle significantly lowers the incidence of PNX and that needles larger than 18 G constitute a risk factor [83,84].

Acquiring more tissue samples is beneficial for future diagnostic purposes, including immunohistochemistry, gene sequencing, and analysis of tumor markers. Increasing the size of the needle, using the coaxial approach, and making many non-coaxial passes through the pleura may increase the volume of samples obtained. In the study conducted by Nour-Eldin et al. [85] there was no increase in the probability of PNX when using the non-coaxial technique. The exact number of passes required for each procedure has not been specified. However, several investigations have indicated a substantial correlation between the number of passes and the occurrence of PNX. The presence of one or two pleural access points did not lead to a higher risk of PNX. However, the risk was shown to be considerably higher when there were three access points. Regardless of the size of the needle used, the main strategy is to minimize the number of passes within the pleural membrane [72,82].

Emphysema substantially raises the possibility of developing PNX. The prevalence of PNX was higher in various subtypes of emphysema compared to non-emphysema in the operable lobe. These findings are consistent with earlier studies, which did not examine specific subtypes of emphysema. Several prior studies either excluded emphysema or ignored it as a risk factor. A chest CT scan can describe the different types of emphysema. These different types of emphysema were strongly associated with the occurrence of PNX after a biopsy [68,86].

Khan et al. concluded that longer puncture duration was related to higher incidences of PNX, and the majority of their patients had lesion sizes larger than 3 cm. There is a common belief that when the pulmonary nodule is smaller, the needle needs to be adjusted more frequently during the procedure. This results in a longer operation time and increases the chance of PNX. When the puncture needle remains in the pleura for a longer period of time, the passageway expands due to breathing movements, leading to a postoperative PNX. Therefore, the operator needs to pay particular attention to the size of the nodules and carefully select the optimal needle trajectory based on the CT image in order to minimize the duration of the procedure [68].

Crossing pulmonary fissure during the needle access showed a statistically significant correlation with the occurrence of PNX. The classification of PNX was determined based on post-intervention axial CT by measuring the extent of retraction of the pulmonary surface. The classifications are as follows: mild PNX, characterized by a lung surface retraction of 2 cm; moderate PNX, characterized by a lung retraction between 2 and 4 cm; severe PNX, characterized by a pulmonary surface retraction of more than 4 cm, rapid accumulation of PNX with mediastinal shift, or the presence of respiratory or circulatory distress [87].

#### 7.1.2. Management Strategies

Najafi et al. conducted a study that revealed that the use of five distinct techniques in CT-guided TTNB can effectively lower the occurrence of pneumothorax to 16% and the need for chest tube insertion to 1%, while maintaining the same level of diagnostic accuracy. The PEARL protocol combines five different procedures: positioning the biopsy side down, removing the needle during expiration, sealing with an autologous blood patch, performing a rapid rollover, and pleural patching [39].

**Figure 4:**
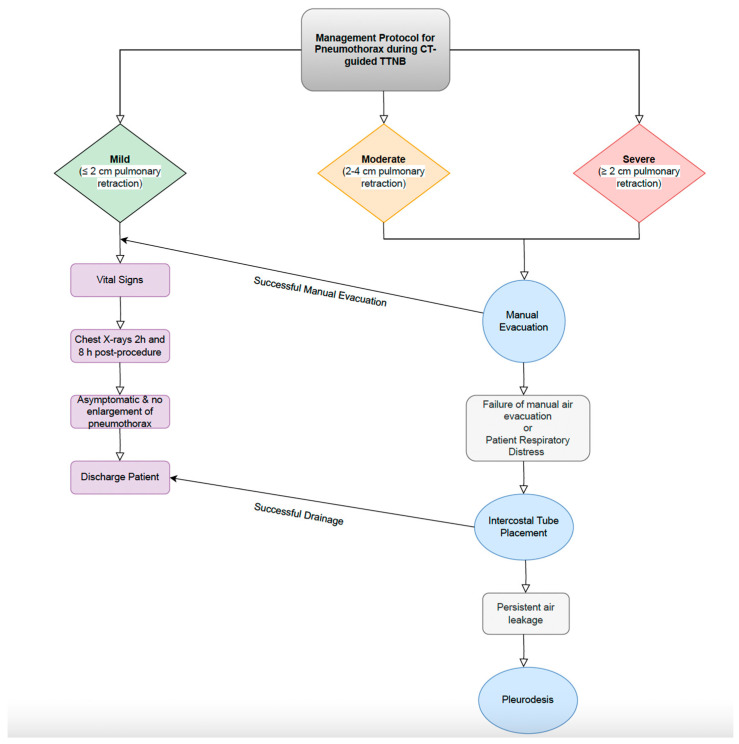
Protocol algorithm for the management of PNX after CT-guided TTNB.

Figure 4 presents the protocol algorithm for management of PNX after CT-guided TTNB.

### 7.2. Pulmonary Hemorrhage

#### 7.2.1. Incidence and Risk Factors

Pulmonary hemorrhage is the second most frequent complication of CT-guided TTNB, occurring between 4% and 27%. Additionally, 27–30% of patients have perilesional ground-glass opacity on CT, indicating bleeding. Hemoptysis affects 4% of people. Higher bleeding rates are associated with smaller lesions (<2 cm), longer needle courses (>4 cm), no pleural effusion, and numerous punctures. Due to technical difficulty, smaller lesions require more needle adjustments and lengthier punctures, which may increase bleeding rates. Lung intrapulmonary bleeding can seal the biopsy tract and prevent pneumothorax. Minor alveolar hemorrhage along the needle route accounts for 86% of pulmonary bleeding. This bleeding appears as ground-glass opacity on CT scans and seldom causes death. Reducing bleeding problems requires careful patient screening and planning before this treatment. Stop anticoagulants per pre-procedural evaluation instructions. Avoid the central and major pulmonary arteries when routing the biopsy needle to avoid bleeding complications. If the patient has significant hemoptysis, halt the surgery [88,89,90].

#### 7.2.2. Management Strategies

Effective management of pulmonary hemorrhage during CT-TTNB requires a combination of preventative strategies, real-time intra-procedural adjustments, and post-procedural vigilance. Early recognition and appropriate intervention are key to minimizing complications and ensuring patient safety [68,91].

Before the procedure, a thorough review of the patient’s medical history, including the use of anticoagulants, antiplatelet agents, and any underlying coagulopathy, is essential. Minimizing the number of biopsy passes can reduce tissue trauma and the likelihood of hemorrhage. Using a smaller gauge needle may decrease the risk of bleeding while still providing adequate tissue for analysis. The use of CT guidance allows for the immediate recognition of hemorrhage during the procedure, often identified as ground-glass opacities. Upon detecting signs of hemorrhage, the biopsy should be paused to assess the extent of the bleeding. In many cases, small, localized hemorrhages will resolve spontaneously without further intervention. Adjusting the patient’s position can help control hemorrhage. For example, placing the patient in a decubitus position with the biopsy side down can utilize gravity to tamponade the bleeding site, limiting blood loss into the airways. Providing supplemental oxygen can assist in maintaining adequate oxygenation if gas exchange is compromised due to hemorrhage. A follow-up CT scan immediately after the procedure can confirm the presence and extent of hemorrhage, helping guide further management. Patients should be closely monitored for signs of respiratory distress, hypotension, or hemoptysis, which may indicate worsening hemorrhage. In cases of significant bleeding, bronchoscopy can be performed to identify and manage the bleeding site. Bronchoscopic techniques such as balloon tamponade, topical application of vasoconstrictive agents, or electrocoagulation may be employed. Endobronchial blockade using a bronchial blocker or balloon catheter can isolate the bleeding segment, preventing blood from flooding the contralateral lung and reducing the risk of aspiration. For severe hemorrhage, fluid resuscitation and blood transfusion may be necessary to stabilize the patient. In rare cases, surgical intervention or embolization of the bleeding vessel may be required [29,92,93].

#### 7.2.3. Air Embolism

Systemic air embolism is a severe complication that can happen during a transthoracic percutaneous lung biopsy. The incidence of an air embolism in symptomatic patients is reported to range from 0.02% to 0.07%. Although a systemic air embolism is rarely encountered, it can lead to a variety of life-threatening cardiac and neurological symptoms. Early and accurate identification of a systemic air embolism can help prevent fatal outcomes [94,95].

Three mechanisms have been proposed for the entry of air into the systemic circulation. Air can enter the systemic circulation through a connection between the pulmonary vein and the atmosphere. The second method occurs through a broncho-venous fistula or another form of connectivity between air-filled areas and pulmonary veins. The third mechanism occurs when air from the pulmonary arterial system enters the pulmonary venous circulation by passing via the pulmonary microvasculature [94].

The clinical symptoms are primarily associated with an air embolism in the coronary or brain circulation. These symptoms include neurological dysfunction (such as focal neurological deficits, seizures, and changes in consciousness) as well as manifestations related to heart ischemia (such as chest pain, low blood pressure, difficulty breathing, abnormal heart rhythms, and cardiac arrest). Importantly, these signs and symptoms can manifest either immediately or a few minutes after the biopsy. In addition, a significant number of patients undergoing a lung biopsy are likely to acquire air emboli and show no symptoms. Considering the unclear characteristics of these clinical modifications, interventional radiologists should perform follow-up CT scans that include the lung in the area where the biopsy was performed, as is commonly done. These scans must additionally include the heart and major blood vessels to identify any presence of air within the blood vessels or heart. [96,97].

If the presence of an air embolism is identified, the optimal course of action involves immediately stopping the procedure, preparing for cardiopulmonary resuscitation measures, warning the rapid response team, positioning the patient in the Trendelenburg position, on their right or left side, administering 100% oxygen, and, if feasible, arranging for placement in a hyperbaric chamber within a period of 4–6 h. The Trendelenburg position, which involves lowering the head below the toes, is recommended for a venous air embolism since it maintains the air within the right ventricular cavity. Yet, evidence from a systematic review [45] indicates that patients with an arterial air embolism positioned in the Trendelenburg position frequently had favorable outcomes. The majority of these cases involved an arterial embolism. In the absence of symptoms, a prudent strategy is putting the patient in the Trendelenburg position as a temporary solution while observing for clinical indicators. The right lateral decubitus position prevents air bubbles from entering systemic circulation and is often used in practice. In instances of arterial air embolism, the left lateral position may offer no advantage as air is already present in the systemic circulation, potentially affecting vital organs such as the brain or heart. In cases of uncertainty regarding the nature of the embolism (venous or arterial), the right lateral decubitus position is frequently recommended for minimizing systemic circulation involvement [98].

Hyperbaric chambers can enhance oxygenation in ischemic tissue, while also decreasing the size of gas bubbles. The chamber can also eliminate the bubbles (nitrogen reabsorption) and prevent the development of cerebral edema [96,99].

#### 7.2.4. Tumor Seeding

Tumor seeding along the biopsy tract is a rare but concerning complication of CT-TCNB of pulmonary nodules. This complication happens when cancerous cells are unintentionally implanted along the needle path, resulting in local recurrence or metastasis [100,101].

The incidence of tumor seeding following CT-guided lung biopsy is very low, estimated to be less than 0.01%. Despite its rarity, the potential consequences of tumor seeding warrant careful consideration, particularly in cases involving malignancies with aggressive behavior or a high propensity for local invasion. The low incidence rate is attributed to several factors, including the relatively short needle path in lung biopsies and the use of coaxial biopsy techniques that minimize needle manipulation [100].

Tumors with high metastatic potential, such as adenocarcinoma and small cell lung cancer, are more likely to seed along the biopsy tract. Adenocarcinomas, in particular, are associated with a higher risk due to their invasive nature and tendency to spread through air spaces. Larger needle gauges and multiple needle passes can increase the risk of seeding by causing more extensive disruption of the tumor and surrounding tissue. [102,103].

Tract ablation, which involves applying thermal energy (such as radiofrequency ablation) or injecting a substance (like ethanol) along the biopsy needle tract after the procedure, has been proposed as a method to destroy any residual tumor cells [102].

The main causes of complications are summarized in Table 3.

## 8. Discussion

### 8.1. Comparison with Other Biopsy Techniques

Fine-needle aspiration (FNA) is a minimally invasive procedure often used for cytological sampling of pulmonary nodules. Compared to CT-TTNB, FNA uses a thinner needle (typically 22–25 gauge), which reduces the risk of complications such as PNX. However, CT-TTNB generally provides a higher diagnostic yield, especially for differentiating between malignant and benign lesions, due to its ability to obtain larger, more intact tissue samples that are suitable for histopathological and molecular analysis. Studies have shown that CT-TCNB has a higher accuracy for diagnosing lung cancer, particularly in cases where architectural features of the tissue are important for diagnosis [56,57,104,105].

Ultrasound-guided percutaneous needle biopsy (US-PNB) is another minimally invasive procedure primarily used for sampling lesions located adjacent to the chest wall. US-PNB is often preferred for its real-time imaging capabilities and lower radiation exposure. This procedure is limited when dealing with deep, central, or small nodules, where ultrasound imaging may not provide adequate resolution [59,106,107].

Bronchoscopy, particularly with the use of advanced tools like radial endobronchial ultrasound (rEBUS) and electromagnetic navigation bronchoscopy (ENB), is a valuable technique for diagnosing pulmonary nodules, especially those that are centrally located. Bronchoscopy is less invasive than CT-TTNB and does not involve the same risks of PNX or hemorrhage associated with transthoracic approaches. However, bronchoscopy’s diagnostic yield diminishes for small, peripheral nodules, where CT-TTNB offers better access and higher accuracy [108].

CT-TTNB vs. Endobronchial Ultrasound-Guided Transbronchial Needle Aspiration (EBUS-TBNA): Endobronchial ultrasound-guided transbronchial needle aspiration (EBUS-TBNA) is a bronchoscopic technique primarily used for sampling mediastinal and hilar lymph nodes or centrally located lung lesions. EBUS-TBNA is less invasive and has a lower complication rate compared to CT-TTNB, but its diagnostic yield decreases for smaller or peripherally located lesions where CT-TCNB excels [109].

Liquid biopsy is a non-invasive method that detects circulating tumor DNA (ctDNA) or other biomarkers in blood samples. While liquid biopsy offers the advantage of being non-invasive and can provide information on tumor genetics and mutation status, it is currently less effective than CT-TTNB for the initial diagnosis of pulmonary nodules. Liquid biopsy is mainly used in monitoring treatment response or detecting recurrence in known lung cancer patients rather than for initial tissue diagnosis. CT-TTNB remains the gold standard for obtaining tissue for histological and molecular analyses necessary for the definitive diagnosis of lung cancer [110].

While each biopsy technique has its own advantages, CT-TTNB stands out for its high diagnostic accuracy, particularly for peripheral pulmonary nodules. It provides adequate tissue samples for comprehensive pathological and molecular analysis, making it a preferred method in many clinical scenarios. However, the choice of technique should be tailored to the patient’s clinical situation, lesion characteristics, and the information needed from the biopsy [111].

### 8.2. Future Directions and Innovations

One promising direction is the integration of molecular imaging techniques, such as positron emission tomography-computed tomography (PET-CT), with CT-TCNB. PET-CT can enhance the visualization of metabolically active (potentially malignant) nodules, allowing for more accurate targeting during biopsy. By combining metabolic and anatomical imaging, PET-CT-guided biopsies may improve diagnostic yield, especially in small or indeterminate nodules where traditional CT alone might not differentiate between benign and malignant tissues [112].

Artificial intelligence (AI) and machine learning algorithms are increasingly being integrated into the planning and execution of CT-TCNB procedures. AI can assist in the real-time analysis of imaging data, helping radiologists predict the optimal needle path and adjust for patient movement or changes in nodule position. Furthermore, machine learning models can analyze large datasets to predict biopsy outcomes and complication risks, potentially allowing for more personalized and safer procedures [113].

Robotic-assisted systems represent another cutting-edge innovation in CT-TCNB. These systems provide greater precision and stability during needle insertion, reducing human error and improving access to difficult-to-reach nodules. Early studies suggest that robotic systems can decrease procedure time and enhance safety by minimizing complications such as PNX. As these technologies develop, they may become a standard part of the biopsy process, particularly for complex cases [114].

## 9. Conclusions

CT-guided transthoracic core-needle biopsy has emerged as a pivotal tool in the diagnosis and management of pulmonary nodules, providing a minimally invasive yet highly accurate method for obtaining tissue samples. This procedure boasts a high diagnostic yield, typically ranging from 85% to 95%, and sensitivity and specificity rates for malignancy detection between 92% and 97%, respectively. Such efficacy surpasses that of many traditional biopsy techniques, particularly in evaluating peripheral lung lesions. The integration of advanced imaging technologies, such as PET/CT fusion imaging and real-time CT fluoroscopy, has significantly enhanced needle placement precision, thereby increasing diagnostic accuracy and reducing the risk of complications. Despite the procedural challenges posed by smaller and deeper lesions, and the potential for complications like PNX and pulmonary hemorrhage, CT-TTNB’s benefits far outweigh these risks when performed with meticulous planning and execution. Furthermore, ongoing innovations, including AI-assisted imaging analysis and robotic-assisted biopsy systems, promise to elevate the safety and effectiveness of this essential diagnostic procedure, making it an indispensable component of modern pulmonary diagnostics.

## Figures and Tables

**Table 1:** Sensitivity and specificity comparison between cone-beam CT and conventional CT for diagnosing malignancy in lung biopsies.

Parameter	Cone-Beam CT (CBCT)	Conventional CT (CCT)
Sensitivity	92% to 97%	93% to 97%
Specificity	96% to 100%	85% to 95%
Advantages	- Real-time 3D imaging for continuous guidance during biopsy - Reduced needle repositioning (up to 84% reduction) - Lower radiation exposure (up to 29% reduction)	- Widely available - Shorter procedural time
Limitations	- Requires specialized equipment - Breath-hold required for minimizing motion artifacts	- More static images requiring multiple scans - Higher radiation exposure

**Table 2:** Complications comparison between cone-beam CT and conventional CT.

Complication	Cone-Beam CT (CBCT)	Conventional CT (CCT)
Pneumothorax	15% to 20%	18% to 25%
Pulmonary Hemorrhage	10% to 24%	15% to 25%
Chest Tube Insertion	1% to 4%	3% to 6%
Hemoptysis	1% to 3%	2% to 5%
Radiation Exposure	10% to 30% lower compared to CCT	Higher compared to CBCT
Needle Repositioning	Reduced by 84% (real-time guidance)	Requires more repositioning, static images
Procedure Time	Typically longer (due to real-time adjustments)	Shorter, more straightforward for simpler cases

**Table 3:** Main causes of complications.

Complication	Causes	Notes
Pneumothorax	- Smaller lesions (<2 cm) - Multiple pleural punctures - Pre-existing emphysema - Deep lesion location	Occurs in 12–45% of cases, with a higher risk in patients with emphysema or multiple needle passes. Chest tube insertion is required in 2–15% of cases.
Pulmonary Hemorrhage	- Needle puncturing blood vessels - Large or central lesions - Anticoagulant use	Hemorrhage may self-resolve in minor cases; serious cases require interventions like bronchoscopy or embolization.
Air Embolism	- Air entering through pulmonary veins - Broncho-venous fistula - Connectivity between air-filled areas and veins	Rare but potentially fatal. Requires immediate identification and treatment.
Tumor Seeding	- Cancerous cells are implanted along the needle path	Very rare (<0.01%) but can occur with aggressive tumors. Larger needles and multiple passes increase the risk.

## Data Availability

No new data were created or analyzed in this study. Data sharing is not applicable to this article.
